# Occult pneumothorax, revisited

**DOI:** 10.1186/1752-2897-4-12

**Published:** 2010-10-29

**Authors:** Hesham R Omar, Hany Abdelmalak, Devanand Mangar, Rania Rashad, Engy Helal, Enrico M Camporesi

**Affiliations:** 1 Departement of Internal Medicine, Mercy Hospital and Medical Center, Chicago, Illinois, USA; 2 Department of Anesthesiology/Critical Care; Tampa General Hospital; Tampa, FL, USA; 3 Florida Gulf to Bay Anesthesiology, Tampa, FL, USA; 4 Critical Care Department, Cairo University Hospital, Cairo, Egypt; 5 Emergency Department, Elagouza Hospital, Cairo, Egypt; 6 Department of Surgery/Anesthesiology, University of South Florida, Tampa, FL, USA

## Abstract

Pneumothorax is a recognized cause of preventable death following chest wall trauma where a simple intervention can be life saving. In cases of trauma patients where cervical spine immobilization is mandatory, supine AP chest radiograph is the most practical initial study. It is however not as sensitive as CT chest for early detection of a pneumothorax. "Occult" pneumothorax is an accepted definition of an existing but usually a clinically and radiologically silent disturbance that in most patients can be tolerated while other more urgent trauma needs are attended to. However, in certain patients, especially those on mechanical ventilation (with subsequent increase of intrapleural air with positive pressure ventilation), missing the diagnosis of pneumothorax can be deleterious with fatal consequences. This review will discuss the occult pneumothorax in the context of 3 radiological examples, which will further emphasize the entity. Because a negative AP chest radiograph can dangerously delay its recognition, we recommend that any trauma victim presenting to the emergency department with symptoms of respiratory distress should be screened with either thoracic ultrasonography or chest CT scan to avoid missing a pneumothorax.

## Introduction

The entity of occult pneumothorax is more frequently recognized nowadays due to the the increasing diffusion of CT scan and thoracic ultrasonography in the evaluation of trauma patients. These diagnostic tools enabled us to detect small abnormalities not clinched by the traditional chest radiograph. The concept of occult pneumothorax has been thoroughly discussed amongst the literature [1-5]. In trauma patients, AP chest radiograph has been traditionally the initial diagnostic imaging study especially if a cervical-collar limits patient mobilization. With advent of the extended FAST examination (Focused Assessment with Sonography for Trauma), most centers now utilize eFAST before the initial screening chest Xray. This review describes the differences between AP chest radiograph and chest CT in early detection of a pneumothorax in a trauma patient.

## Definition

Occult pneumothorax is a pneumothorax that was not suspected clinically nor was evident on the plain radiograph but rather identified on computed tomography scan.

**Figure 1:**
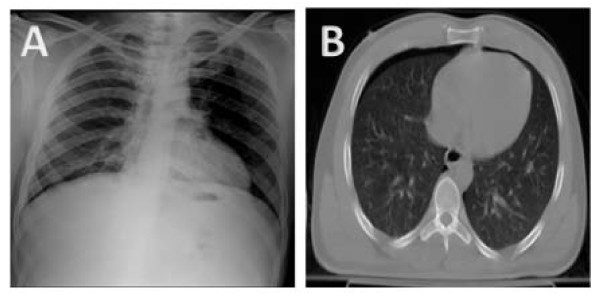
**AP chest X-ray revealing no evidence of pneumothorax (Panel A)**. CT chest performed immediately after X-ray revealed right sided pneumothorax (Panel B).

Due to the increased utilization of CT chest and thoracic ultrasonography as the initial screening tests for thoracic and abdominal trauma, occult pneumothorax has been common. Most nonradiologists diagnose pneumothorax based on the visualization of a superior-lateral visceral pleural stripe on the upright chest radiograph. This is however not feasible in the supine chest radiograph unless there is a sizable pneumothorax. Unfortunately, because of clinical concerns in trauma patients regarding cervical spine immobilization, initial imaging in seriously injured patients typically consists of a supine AP chest X-ray that is insensitive for detecting pneumothorax. As illustrated in figure 1 after an initial AP chest radiograph failed to show evidence of pneumothorax, a CT chest performed immediately after the chest Xray revealed right-sided pneumothorax.

## Incidence

The reported incidence of occult pneumothorax varies widely between 3.7% in injured children presenting to an emergency department to 64% in intubated multi-trauma patients[6-8]. However, most publication agreed with an average incidence of 5% for all trauma patients. This highlights the inadequacy of the supine AP chest X-ray as compared to chest CT in detection of a post-traumatic pneumothorax. The incidence depends on the type of trauma, whether blunt or penetrating. In patients with blunt trauma the incidence of occult pneumothorax ranges between 2% and 15% depending on whether all patients in a given registry, or only those that underwent CT, are included. This value may increase when the trauma team does the interpretation of the chest radiograph. In a retrospective study conducted by Wilson et al,[9] 1881 consecutive blunt trauma patients over a 102 month period were included. 307 patients developed pneumothoraces of which 68 were occult (22%). The frequency of occult pneumothoraces in victims of penetrating trauma approaches 17%. This was described in a recent level-III study of 5552 admissions at Grady Memorial Hospital [10].

Moreover, the reported proportion of pneumothoraces that are occult compared with those actually present on supine AP chest radiograph is variable and ranges from 29% to 72% [1-4,11]. This variability is probably due to the fact that in some studies it is not always a certified and well-experienced radiologist who is responsible for making the diagnosis, which is the most precise way to identify the true incidence. A 17 month prospective study performed by Ball et. al.[12] concluded the incidence of occult pneumothorax to be as high as 76% when the radiographs are interpreted by the trauma team. This is higher than previously reported values in retrospective studies and is likely due to the difficult conditions in which the trauma team functions. Another factor that affects the variability in calculating the incidence of occult pneumothorax is whether anteroposterior chest radiograph or erect chest Xray is used. The reported sensitivity of erect chest X-ray vs. AP chest X-ray in detecting occult pneumothorax is 92% and 50% respectively. This highlights the inadequacy of supine AP chest radiography for detecting pneumothorax and the requirement for a thoracic CT or ultrasonography to rule out the diagnosis in trauma patients.

## Why Occult?

In the upright position, the classic sign for the diagnosis of pneumothorax is the visceral pleural line, which is visible as a thin curvilinear opacity along the lung and is separated from the chest wall by air in the apical pleural space. This sign is rarely identifiable on radiographs of supine patients unless there is a sizable pneumothorax. Small to moderate sized pneumothoraces may easily escape detection in that position.

In the supine patient, the least dependant pleural spaces are the anteromedial and subpulmonic recesses. Accumulation of air is expected in these 2 spaces initially with further extension laterally and apically as air volume increases or as the patient position becomes more upright. Therefore free air travels first to the highest region of the thorax; the cardiophrenic region. Larger volumes of free air then extend to the subpulmonic region inferiorly and to the anteromedial region superiorly [13]. These sites are easily missed on the conventional anteroposterior chest radiograph and indicate that the gold standard for ruling out pneumothorax is a thoracic CT scan.

Other than the body position, another determinant influencing the distribution of pleural air is an alteration in lung recoil due to consolidation or adhesion [14]. For example, in cases of postoperative left lower lobe collapse due to single lung ventilation (if the tube is advanced into the right main bronchus); the development of pneumothorax in these patients will usually have a posteromedial distribution [14]. Similarly, in patients with obliterated pleural areas due to adhesions, air will be excluded from these spaces.

Occult pneumothorax is concerning because of the risk of rapid progression to tension pneumothorax with positive pressure ventilation especially in trauma patients who have diminished cardiopulmonary reserve. Furthermore, clinical respiratory distress in these patients may be masked by sedation and concomitant respiratory support.

## Radiographic clues for suspicion of occult pneumothorax

In supine or semi-erect patients, pneumothoraces do not appear in their classically described location over the apex and lateral to the lung. The insensitivity of the AP chest radiograph in the diagnosis of pneumothorax in supine patients has invited research looking for other possible clues for suspecting the diagnosis. The clues that help in raising the suspicion of pneumothorax in the AP chest radiograph if the classic signs are not evident include: [15-19]

1. The high visibility of the cardiophrenic sulcus.

2. The deep sulcus sign which represent lucency of the lateral costophrenic angle extending toward the hypochondrium

3. Depression of the ipsilateral hemidiaphragm due to increased intrapleural pressure.

4. Double-diaphragm appearance due to air outlining of the anterior costophrenic angle and aerated lung outlining the diaphragmatic dome.

5. Improved sharpness of the cardiomediastinal border with a distinct cardiac apex due to anteromedial collection of air, which may appear as lucency. A sign known as crisp cardiac silhouette.

6. Increased sharpness of the pericardial fat pads, which become rounded and lobulated in the presence of air in the pleural space because they are no longer flattened by contact with the adjacent lung.

7. Visible inferior border of a collapsed lower lobe. A thin, sharp line may be detected which represents the inferior surface of the lung (visceral pleura) elevated and outlined by the inferior pneumothorax.

8. A band of air in the minor fissure bounded by two visceral pleural lines.

9. Visible lateral edge of the right middle lobe due to medial retraction in the presence of anterior pneumothorax.

Knowledge of these clues is mandatory for suspecting pneumothorax on AP chest X-ray. Radiologists, ER and critical care physicians should be aware of these signs to avoid missing a pneumothorax.

## Is occult pneumothorax predictable?

**Figure 2:**
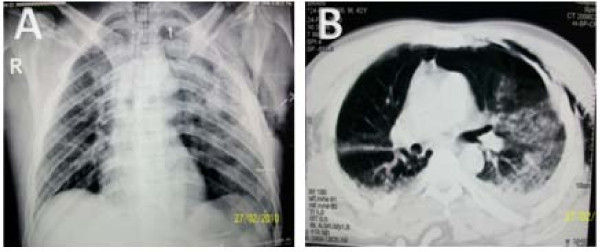
**AP chest X-ray revealing evidence of bilateral lung contusions and left subcutaneous emphesema (Panel A)**. Chest CT confirmed both the lung contusions and the subcutaneous emphesema and demonstrated a left sided pneumothorax not initially appearing on the anteroposterior chest Xray (Panel B).

Yes, there are several clinical markers that can predict for an increased incidence of pneumothorax even if not detected on the initial anteroposterior chest radiograph. These markers should be well known to emergency and ICU physicians for early suspecting the diagnosis. A level III retrospective study [20] with a purpose to determine the incidence, predictors, and outcomes for occult pneumothorax after trauma concluded that the presence of subcutaneous emphysema, pulmonary contusions, rib fractures and female sex were crudely associated with the presence of occult pneumothorax with an odds ratio of 5.47 for subcutaneous emphesema, 3.25 for pulmonary contusions and 2.65 for rib fractures. Although only 16% of patients with occult pneumothorax had subcutaneous emphysema, 98% of the patients with subcutaneous emphysema had an underlying pneumothorax whether overt (82%) or occult (16%). Therefore subcutaneous emphesema has a very high specificiy for diagnosis of occult pneumothorax but its absence is insufficient to rule out the diagnosis. Four years later, on a subsequent prospective level-II study [12] performed by the same group, only subcutaneous emphysema remained the only independent risk factor that can predict occult pneumothorax. Other risk factors including patient's age, mechanism of injury, intubation status in the emergency department, seat belt use, GCS score and the presence of pre-exisisting pulmonary comorbidities showed no significant predictive value for occult pneumothorax. We suggest that any trauma patient presenting with subcutaneous emphesema, pulmonary contusion or rib fractures should be further evaluated with CT chest to exclude any underlying pneumothorax not visible on the regular chest radiograph. Figure 2 well demonstrates the predictability of occult pneumothorax. The presence of lung contusions and subcutaneous emphesema has prompted further evaluation with CT chest, which revealed a pneumothorax.

## Truly Occult or Missed Pneumothorax?

In a retrospective review of 44 severely injured patients identified with OPTX, 3 groups of radiologists reviewed the images for signs of pneumothorax [21]. The first group of radiologists which comprised 3 board-certified radiologists confirmed the presence of actual pneumothorax in 9 patients. The second and third group, which comprised a single board certified radiologist, each confirmed the presence of actual pneumothorax in 10 and 4 patients respectively. The deep sulcus sign was by far the most common "missed" sign (75-90% of the missed pneumothorax depending on the group). Only 1 sharpened cardiac silhouette and an actual missed pleural line were also observed as shown in table 1.

**Table 1:** Secondary Signs of PTXs Identified on Supine AP chest Xray

	Group 1	Group 2	Group 3
Deep sulcus	7	9	3

Crisp cardiac silhouette	1	1	0

Pleural line	1	0	1

Adapted from Ball et al. from reference [21].

Furthermore, these additional PTX signs observed on retrospective review were not identified consistently in a given patient across radiologist groups. When one considers this inconsistency among highly trained radiologists, with the reality that supine AP chest X-ray are typically first interpreted by non-radiologist trauma surgeons, the utility of this imaging modality is unclear. These secondary signs are often quite subtle and are rarely used by the clinicians making acute therapy decisions in the trauma bay. As previously mentioned, in the 17-month prospective level-II study, the incidence of occult pneumothoraces appeared as high as 76% when interpreted by the trauma service at the time of admission [12]. Although the team may have good interpretive skills, they do not routinely have the luxury of prolonged interpretation times, a perfectly lit environment and premium digital monitors.

**Figure 3:**
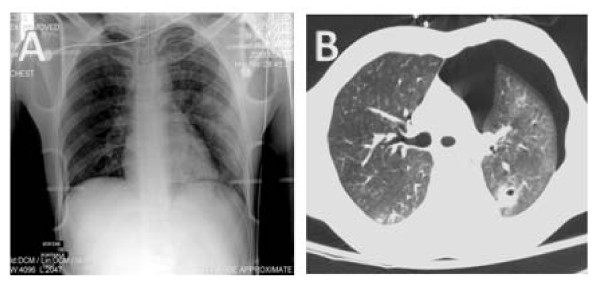
**AP chest X-ray of the intubated patient, illustrating diffuse air space opacities in the left lower lung field (Panel A)**. Underlying pneumothorax was suggested because of a visible pleural stripe in the lung apex and a visible cardiophrenic sulcus. Chest CT scan illustrating a left-sided pneumothorax with underlying lung collapse (Panel B).

Figure 3 well illustrates how pneumothorax can present with subtle radiographic finding and therefore leading to a missed diagnosis. Notice the visible cardiophrenic sulcus and the apical pleural stripe in the AP chest radiograph. These were missed during interpretation of the Chest X-ray and could have lead to deleterious effects if passed un-noticed.

## Thoracic ultrasonography for early detection

Although CT lung scanning is presently considered to be the standard diagnostic imaging for Pneumothorax, it has some disadvantages, including the need for patient transportation (which is not usually feasible in the unstable patient) and high doses of radiation. Lung ultrasonography has emerged in the past decade as a new and sensitive technique in the evaluation of respiratory diseases with a sensitivity of detecting pneumothoraces ranging from 92% to 100% among patients with blunt injuries [22-25]. Other advantages include the fact that it can be easily and quickly performed at bedside by a wide range of "sonographers," such as trauma, emergency, and critical care physicians [25]. The possible role played by chest ultrasonography in detecting the size and extension of a PTX is a challenging task which would allow the emergency department physician to take interventional decisions, such as the positioning of a chest tube, without wasting time. Potential pitfalls for thoracic ultrasonography such as the presence of pleural adhesions and emphysematous bullae are less common in trauma patients. Therefore ultrasonographic evaluation of the thorax should be performed during the primary survey as a part of the eFAST examination for trauma patients[26]. This will identify a significant number of radio-occult Pneumothoraces and allow for sonar-guided interventions without exposing the unstable patients to the hazards of transportation and ionizing radiation.

## Management

Clinicians appear to have greater difficulty deciding the appropriate therapy in patients with occult pneumothorax. The choice between close observation vs. early intercostal tube placement is still debatable, with reports of more complications from chest tube placement than from the pnemothorax, as long as the team remains aware of the pneumothorax. The traditional management of the majority of post-traumatic pneumothoraces detected clinically, or on chest X-ray, has been the placement of a chest tube. Tube thoracostomy is associated with up to 22% rate of major complications [27]. These include insertional (intercostal artery or intraparenchymal lung injuries), positional (requiring reinsertion) and infective (empyema or wound infection) issues [28-33]. A suggested algorithm for diagnosis and management of occult pneumothorax has been thoroughly described by Ball et. al. This algorithm utilized early thoracic ultrasound to limit CT over-usage [34].

In some patients, expert opinions support that close observation is safe, provided that the patient is not mechanically ventilated and that the pneumothorax is not increasing in size [3,4,35,36]. The importance of therapy is more relevant in the intubated patient on positive-pressure ventilation, as they are at increased risk of complications. However, the literature does not definitively identify which subset of patients receiving mechanical ventilation should receive or safely avoid tube thoracostomy.

Some authors believe that the risk of progression of a known pneumothorax to a tension pneumothorax is significant and that prophylactic chest tube placement for any patient with an occult pneumothorax subjected to positive-pressure ventilation is necessary [10,37,38]. And since the size of the initial occult pneumothorax is not predictive of progression or formation of a tension pneumothorax, therefore it cannot be used as a guide for placing a chest tube [30]. With all these conflicting results, the appropriate management for occult pneumothorax is still unsettled.

## Conclusion

In conclusion thoracic CT scan is the "gold standard" for early detection of a pneumothorax, and is the imaging modality of choice for seriously injured blunt trauma patients. In centers utilizing eFAST technology for trauma victims, this should be the initial diagnostic modality. Its high sensitivity approaching 100%, its availability at bedside without the need for transporting an unstable patient and its low profile for radiocarcinogenesis makes it an ideal tool. If not feasible, then we recommend that any trauma victim presenting to the emergency department with symptoms of respiratory distress should be offered a chest CT scan even if the chest X-ray showed no abnormalities.

This represents an in-depth illustrated review that will help understanding the entity of occult pneumothorax. The review is instructive for several reasons. First, it highlights the importance of the X-ray being interpreted by both a qualified radiologist and the ER or ICU physician to avoid missing an existing pneumothorax. Second, it demonstrates the predictability of pneumothorax in cases presenting with surgical emphesema, rib fractures or lung contusions. Third, it lists the different clues in the AP chest radiograph performed for the supine patient that can induce suspicion of an underlying pneumothorax if the classic signs are not present. Fourth, it emphasizes how the delay in diagnosing pneumothorax can dangerously affect the outcome and fifth, it emphasizes the importance of performing a thoracic ultrasound or a computed tomography of the chest following an initial negative AP chest radiograph in trauma victims with signs of respiratory distress especially those who will receive positive pressure ventilation.

## Competing interests

The authors declare that they have no competing interests.

## Consent

Written informed consent was obtained from the patient's relatives for publication of this review article. A copy of the written consent is available for review by the Editor-in-Chief of this journal

## Authors' contributions

HO was responsible for literature search and drafting the manuscript and providing the explanatory figures. HA, EC and DM, RR and EH have made critical revisions to the manuscript. All authors have read and approved the whole manuscript.
